# Development of Antimicrobial PLA Composites for Fused Filament Fabrication

**DOI:** 10.3390/polym13040580

**Published:** 2021-02-15

**Authors:** Zachary Brounstein, Chris M. Yeager, Andrea Labouriau

**Affiliations:** 1 Los Alamos National Laboratory, Los Alamos, NM 87545, USA; zrbrounstein@lanl.gov (Z.B.); cyeager@lanl.gov (C.M.Y.); 2 Department of Nanoscience and Microsystems Engineering, University of New Mexico, Albuquerque, NM 87131, USA

**Keywords:** fused filament fabrication, FFF, additive manufacturing, AM, 3D-printing, PLA, ZnO, TiO_2_, polymer composites, PEG

## Abstract

In addition to possessing the desirable properties of being a biodegradable and biocompatible polymer fabricated from renewable resources, poly (lactic acid) (PLA) has useful mechanical and thermal attributes that has enabled it to be one of the most widely-used plastics for medicine, manufacturing, and agriculture. Yet, PLA composites have not been heavily explored for use in 3D-printing applications, and the range of feasible materials for the technology is limited, which inhibits its potential growth and industry adoption. In this study, tunable, multifunctional antimicrobial PLA composite filaments for 3D-printing have been fabricated and tested via chemical, thermal, mechanical, and antimicrobial experiments. Thermally stable antimicrobial ceramics, ZnO and TiO_2_, were used as fillers up to 30 wt%, and poly (ethylene glycol) (PEG) was used as a plasticizer to tune the physical material properties. Results demonstrate that the PLA composite filaments exhibit the thermal phase behaviors and thermal stability suitable for 3D-printing. Additionally, PEG can be used to tune the mechanical properties while not affecting the antimicrobial efficacy that ZnO and TiO_2_ imbue.

## 1. Introduction

The increasing awareness that traditional plastics derived from petrochemicals can accumulate in the environment and elicit long-lasting deleterious effects have galvanized efforts to develop and bring to market replacement polymers that are more amenable to recycling and upcycling. One such polymer, poly (lactic acid) (PLA), has garnered much interest due to its lifecycle and material properties. PLA can be synthesized from renewable resources and is biodegradable when composted at high temperatures and humidity [1,2,3]. Its chemical, thermal, and mechanical properties also lend itself numerous advantages: it is biocompatible, easily processable, and has a high strength and Young’s modulus, even matching or exceeding that of polystyrene and poly (ethylene terephthalate), two of the world’s most common plastics made from petrochemicals [4,5,6,7,8]. Because of this, PLA is used within industries spanning medicine, food and agriculture, and packaging.

Due to these beneficial characteristics, neat PLA is also one of the most popular polymers used in additive manufacturing (AM), especially within the domain of fused filament fabrication (FFF), a 3D-printing technology [6,9]. FFF works by heating thermoplastic filaments past their glass transition temperature or melting point and extruding the softened material through a nozzle onto a bed, where the filament cools down slow enough to adhere to the layers placed below and above it. Despite past advances in FFF technology, a barrier towards future growth and expanded applications is the relatively small amount of feedstock polymers currently available for use [10,11].

To enhance the existing materials properties of PLA, and introduce new characteristics, many groups have investigated making composites, such as incorporating graphene-based components to increase the thermomechanical stability or imbue electrical conductivity [12,13,14] or blending PLA with other polymers to make a more mechanically and thermally stable material [15,16,17,18,19,20]. Although PLA is generally more resistant to biodegradation that other natural polymers [8,21], some applications such as transporting agricultural products or medical equipment demand more extensive antimicrobial properties. To meet this demand, previous work has demonstrated that PLA can be combined with bactericides, such as the ceramics ZnO and TiO_2_. However, composites of this form have not been thoroughly explored in the realm of FFF technology [22,23,24,25,26,27,28]. Another issue to take note of is that while PLA/ceramic composites have been investigated for some time, much of the current research is focused on utilizing nanoparticles. Beyond the potential toxicity attributed to ZnO and TiO_2_ nanoparticles [29,30,31,32], many of these studies present varied results with regards to material properties [22,24,33,34]. Breaking with this current trend, we contend that PLA composites incorporating micron-sized or agglomerated nanoparticle forms of either ZnO, TiO_2_, or both, could address some of these issues.

Another challenge encountered during the development of PLA/ceramic composites for FFF technology is the stiffness and brittleness imparted on the material when ceramics are incorporated [35]. To remediate this, further material advances can be made in PLA composites through inclusion of poly-(ethylene glycol) (PEG), another biocompatible and biodegradable polymer, which acts as a miscible plasticizer in PLA and can be added in quantities up to 20 wt% without causing phase separation [15,36,37]. As an added benefit, PEG can be conjugated to target molecules (e.g., drugs) or easily modified to produce specific attributes [37,38,39,40,41]. Due to these properties, incorporating PEG into filaments for 3D-printing can lead to the development of a wide range of extremely useful multifunctional composite filaments with tunable material properties.

In this work, tunable, multifunctional antimicrobial composite PLA filaments were developed and characterized for FFF technology. Antimicrobial functionalities were successfully added to PLA feedstock by uniformly dispersing varying amounts of ceramic ZnO and TiO_2_ fillers in a PLA/chloroform solution. Additional chemical, thermal, and mechanical functionalities were imbued with the addition of different molecular weight PEG at 10 wt% to the PLA/chloroform solution. Each specimen was thoroughly combined and the resulting mixture was cast and subsequently pelletized. Smooth and homogenous printable filaments were created by extruding the composite pellets. The chemical structure of the filaments was investigated to ensure homogeneity, thermal stability was determined to characterize degradation and range of feasibility, phase transitions were reviewed to ensure allowable use in FFF, mechanical limits were determined to understand structure-property relationships, and antimicrobial efficacy was tested.

## 2. Materials and Methods

### 2.1. Materials

PLA 4043D pellets were supplied by Filabot. TiO_2_ powder was supplied by Alfa-Aesar. Chloroform, ZnO powder, and PEG (1 k, 2 k, and 10 k) were purchased through Thermo Fisher Scientific.

### 2.2. Feedstock and Filament Fabrication

The overall process for fabricating filament feedstocks was similar to the work previously described for acrylonitrile butadiene styrene (ABS) [42]. PLA pellets were combined with the antimicrobial agents (TiO_2_, ZnO) and PEG to achieve the desired weight ratios. Chloroform was added to PLA to achieve a 1:5 *w*/*w* solution. The chloroform-PLA mixture was manually stirred for 30 s before being placed in an oven at 25 °C for 16 h to fully dissolve the PLA. If added, fillers were then suspended in the solution. To decrease the viscosity and allow for more homogenous stirring, an additional 15 mL of chloroform was added and the resulting slurry was thoroughly combined using a planetary mixer at 2000 rpm for two minutes. The mixture was then cast onto a clean Teflon-coated plate and left to dry in a fume hood for at least 12 h. This allowed excess chloroform to evaporate from the casted composite, while retaining enough malleability in the composite to cut it into 1 cm × 1 cm squares.

The composite squares were placed in a vacuum oven at 65 °C and 80 kPa negative pressure for at least 24 h, to fully evaporate the remaining chloroform. The dried samples were then fed into a Filabot EX2 extruder heated to 175 °C and set to its maximum extrusion rate (2 lb/h). A container of room temperature DI water was placed a meter below the extruder. Thus, gravity pulled the filaments into the water, which cooled and hardened the samples and produced naturally coiled filaments. The filament samples created using this method and their compositions are listed in Table 1.

### 2.3. Characterization Techniques

#### 2.3.1. Chemical Structural Characterization

Chemical structural characterization of the PLA filaments was carried out using Fourier transform-infrared spectroscopy (FTIR) and micro X-ray fluorescence (MXRF). A Nicolet iS50 FT-IR instrument was used to assess polymer functional groups by measuring absorbance from 525 to 4000 cm^−1^ (32 scan increments) and subtracting the background values. A Bruker M4 Tornado MXRF instrument was used to generate elemental color maps of filament cross sections to examine ceramic distribution. The acquisition parameters included an X-ray tube operating at 50 kV and 200 µA, a spectrometer operating at 40 keV and 130 kcps, a spot size of 20 µm, a dwell time of 5 ms per pixel, and a step size of 10 µm (cross section) by 20 µm (top down).

Particle size distribution of the ZnO and TiO_2_ ceramic fillers was assessed using a Horiba particle size analyzer LA-960. Three different procedures were used in this determination: (1) mixing the individual fillers in a vial of water and circulating the slurry in the reservoir of the instrument; (2) mixing the individual fillers in a vial of water and pipetting the slurry in the reservoir without circulation; and (3) placing the individual fillers in the reservoir with circulation and 30 s of ultrasonication employed. All three procedures were performed in triplicate. During the analysis using the software with instrument, refractive indices of 2.00 and 2.75 were used for ZnO and TiO_2_, respectively.

#### 2.3.2. Thermal Characterization

To investigate thermal properties of the PLA filaments, differential scanning calorimetry (DSC) and thermogravimetric analysis (*T*_g_A) were performed. DSC was performed using a TA Instruments DSC Q20 series instrument where each sample had a mass between 5 and 10 mg and the protocol ran under nitrogen with a flowrate of 5 mL/min. The protocol started by heating from room temperature to 200 °C at a rate of 5 °C/min, then cooling to 120 °C at a rate of 10 °C/min, a slower cooling to 80 °C at a rate of 1 °C/min, a cooling to 25 °C at a rate of 10 °C/min, and ending with heating to 200 °C at a rate of 5 °C/min. The first heating step in the procedure (to 200 °C) was performed to measure the melting point temperature, *T*_m_, and latent heat of fusion, Δ*H_f_*, of the different PLA composites. To simulate the thermal environment of a realistic 3D-printed part, which includes heating in the nozzle, cooling on a bed, and then used, the glass transition temperature, *T*_g_, was obtained upon the second heating. In addition to determining material properties, slowing the cooling rate at 120 °C to 1 °C/min was done to help induce crystallization, although it was to no avail. Using the latent heat of fusion, the PLA weight percent of the sample, *w*, and the theoretical enthalpy of formation $\Delta H_{f}^{\prime }$, taken as 93.0 J/g [43], the percent crystallinity, *X_c_*, of each sample was determined using Equation (1). Based on previous experiments and comparative measurements, the error associated with this instrument is under 3%.

(1) $$X_{c}=\frac{\Delta H_{f}}{w\Delta H_{f}^{\prime }}\times 100\%$$

*T*_g_A was performed using a TA Q50 Series instrument, where each sample had a mass of 10 ± 1 mg and was heated from room temperature to 600 °C at heating rates of 5, 8, 10, 13, and 15 °C/min under a nitrogen flowrate of 40 mL/min. The mass percent and mass derivative curves were measured to evaluate thermal stability, where the degradation temperature, *T*_d5%_, was taken when the sample had lost 5% of its initial mass, and the decomposition temperature, *T*_dMax_, was taken as the maximum of the mass derivative curve. Pyrolysis kinetic parameters were also assessed using Equation (2), known as the Coats–Redfern equation [44,45,46,47]. Using the mass-loss percent, *α*, taken from the *T*_g_A experiments, the absolute temperature, *T*, along with the heating rate, *β*, it is possible to estimate the thermal activation energy, *E*, and pre-exponential factor, *A*. This is done by assuming a reaction model, *g*(*α*), and plotting *ln*(*g*(*α*)/*T*^2^) against 1/*T*, which in theory produces a linear relationship with slope *–E/R* and intercept *ln*(*AR*/*βE*). This method assumes that a single mechanism is responsible for the thermal decomposition. As such, pyrolysis kinetic parameters were calculated using different heating rates, reaction models, and temperature intervals. Based on comparative measurements and calibration testing from the manufacturer, the error associated with this instrument is under 1%.

(2) $$ln\left(\frac{g\left(\alpha \right)}{T^{2}}\right)=ln\left(\frac{AR}{\beta E}\right)-\frac{E}{RT}$$

#### 2.3.3. Mechanical Characterization

Mechanical characterization was performed using tensile testing. Each sample was cut to 10-cm length and placed in an Instron 4340B instrument, where filament ends (3 cm) were clasped, leaving a 4 cm section in the middle of the filament. At a constant rate of 8.333 × 10^−3^ mm/min, the filaments were stretched until breaking. At least 3 replicates of each filament material were tested. Engineering stress versus strain curves were generated, and the Young’s modulus, as well as the maximum stress and strain values, were determined. Based on comparative measurements and calibration testing from the manufacturer, the error associated with this instrument is under 1%.

### 2.4. Antimicrobial Efficacy

Antimicrobial efficacy of the different PLA composites was assessed by incubating filaments (0.5–2 g) in soil at 30 °C for 1 month. Soils were moistened to saturation every 2–3 days. Two trials were run. The first trial used one of every type of filament produced and the second trial used PLA and PLA/ceramic 90/10 composite filaments with or without the addition of 10 wt% gelatin to the soil. Gelatin was added as a possible means to stimulate growth of microorganisms capable of degrading PLA [48]. Following the incubation period, samples were removed from the soil and partially cleaned and inspected in a series of steps. First, small dirt particles were partially dislodged from dry filaments using a vortex mixer. Second, each specimen was submerged individually in sterile water and vortex mixed. After this washing, a crystal violet solution was used to stain the samples. Following this, a 30% acetic acid solution was used to partially de-stain the filaments. At each step, filaments were visually inspected by stereo microscopy using a Leica EZ4E to assess the extent of pitting/degradation and microbial colonization.

## 3. Results and Discussion

The particle size distribution analysis produced three parameters of interest for each ceramic filler: D10, D50, and D90, where 10%, 50%, and 90% of the population fall below this value, respectively. These parameters, shown in the Supplementary Information as Tables S1 and S2, revealed that 80% of the broken up ZnO and TiO_2_ particles were approximately between 1 and 7 microns. It should be noted that the ceramic fillers were not broken up via ultrasonication or any other method prior to their incorporation in the PLA/chloroform solution. This means that the particle sizes used in the composite filaments were agglomerated into sizes larger than the nanoscale.

**Figure 1:**
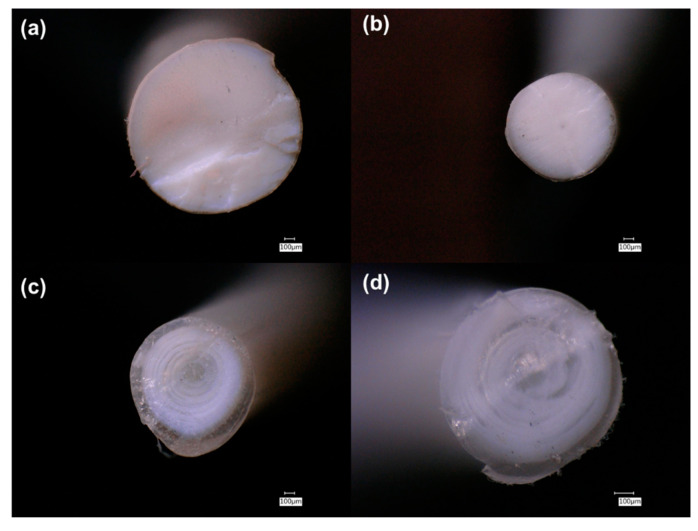
Digital microscopy cross-section images of: (**a**) PLA/TiO_2_ 90/10; (**b**) PLA/ZnO 90/10; (**c**) PLA/TiO_2_/PEG2k 80/10/10; and (**d**) PLA/ZnO/PEG2k 80/10/10.

**Figure 2:**
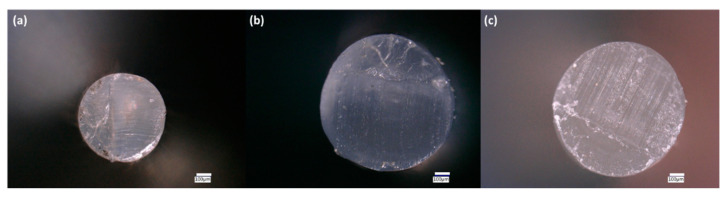
Digital microscopy cross-section images of: (**a**) PLA/PEG1k 90/10; (**b**) PLA/PEG2k 90/10; and (**c**) PLA/PEG10k 90/10.

Examination of the cross-sections of the filaments using a VHX 6000 digital microscope revealed several interesting features (Figure 1 and Figure 2). The first row of Figure 1a,b shows PLA/ceramic filaments, and while they appear homogenous in the core, a thin shell or crust can be seen. This becomes more apparent in the second row of Figure 1c,d which shows PLA/ceramic/PEG2k filaments. A similar core/shell structure is seen but a much thicker crust is observed. Cross sections of PLA/PEG filaments are shown in Figure 2, which does not show any observable crust. As discussed later on, the ceramic composite filaments exhibit a greater crystallinity with PEG than without PEG (shown in the Supplementary Information Table S3). Based on this data and from the microscopy images in Figure 1 and Figure 2, it appears that the crust is different from the interior of the filaments. Additionally, because DSC data (shown later and Table SI3) demonstrates that the filaments are semi-crystalline, it can be inferred that there are amorphous-rich and crystalline-rich regions.

### 3.1. Chemical Structural Characterization

#### 3.1.1. FTIR

**Figure 3:**
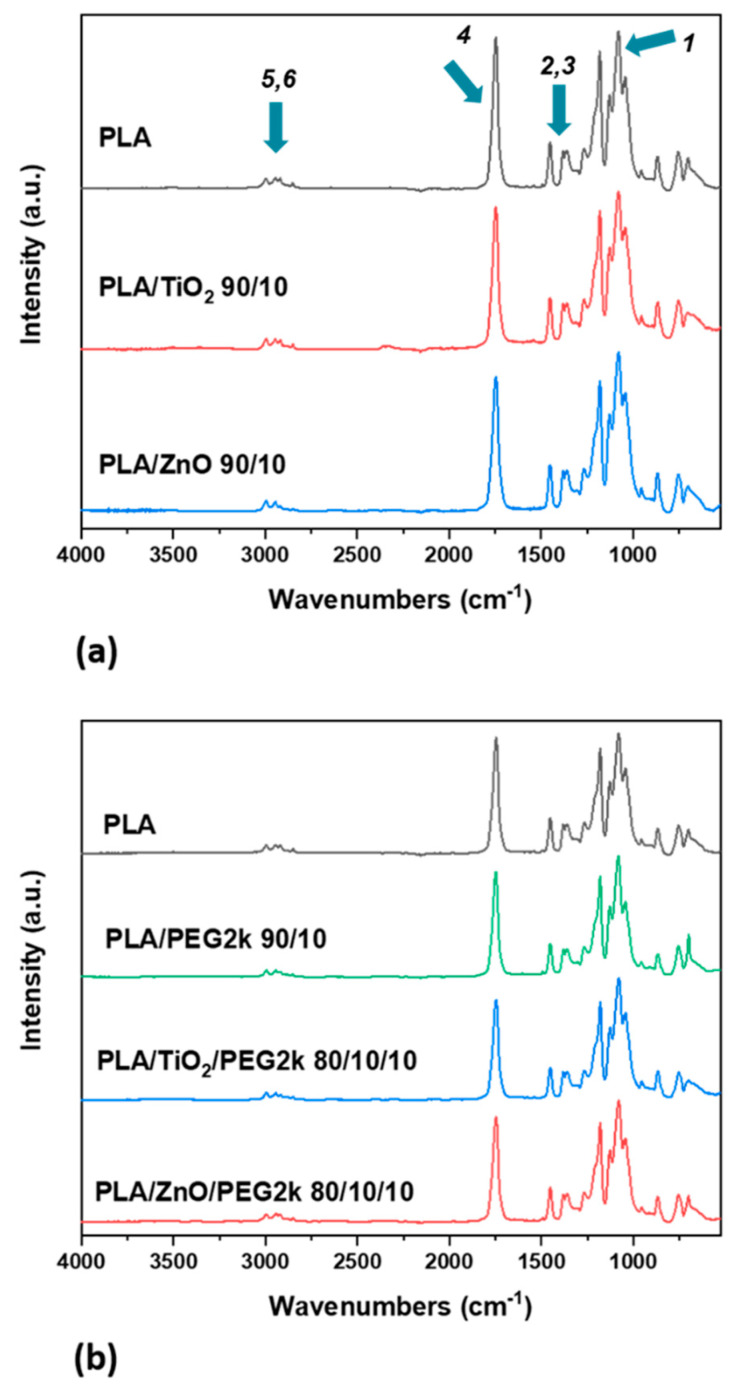
FTIR spectra of PLA (**a**) with 10 wt% ceramic fillers and (**b**) with 10 wt% PEG. The arrows correspond to peaks that are detailed in Table 2 below.

The chemical structure of the different PLA composite filaments (Table 1) was assessed using FTIR. Figure 3 shows the FTIR absorbance spectra of a 100% PLA filament compared to PLA composites with 10 wt% ceramic fillers, 10 wt% PEG, or a combination of both. There is a distinctive band at 1757 cm^−1^ indicating a carbonyl group from PLA and one at 2882 indicating -CH_2_ stretching from PEG [7,49,50]. Other notable peaks in Figure 3 include -CH- symmetric and asymmetric bending from 1130 to 1270 cm^−1^ [51]. Because PEG only makes up 10 wt% of the filaments, its typical peaks are not strongly exhibited. This has been observed in previous research, where composites up to 10 wt% PEG resemble neat PLA [50,52]. The major FTIR peaks associated with the functional groups of PLA are detailed in Table 2. Overall, the FTIR spectra do not show evidence for the formation of new bonds or moieties, thus demonstrating that no chemical reactions occurred between PLA, PEG, or ceramics and that the filament is a true composite.

#### 3.1.2. MXRF

**Figure 4:**
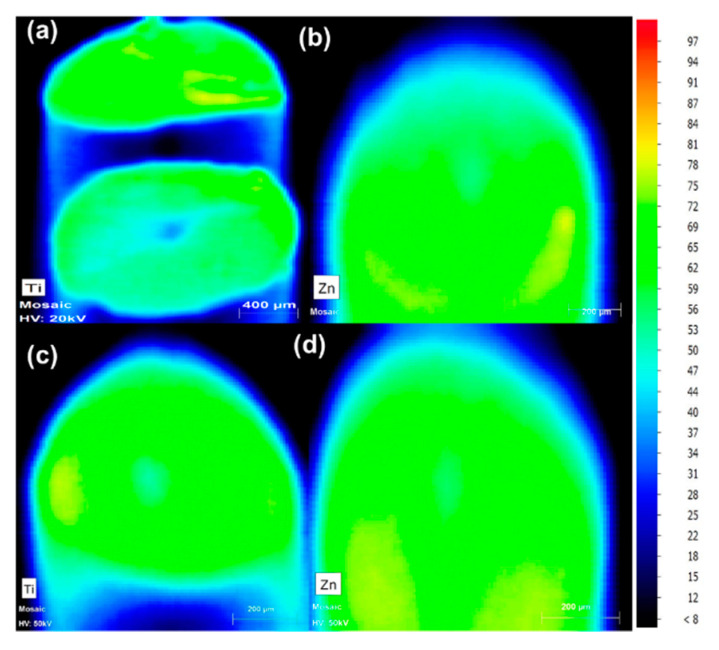
MXRF color mapping of: (**a**) Ti in PLA/TiO_2_ 90/10; (**b**) Zn in PLA/ZnO 90/10; (**c**) Ti in PLA/TiO_2_/PEG2k 80/10/10; and (**d**) Zn in PLA/ZnO/PEG2k 80/10/10. Increasing amounts of the indicated element are colored from blue to green to red.

To ensure that the ceramics were successfully incorporated, MXRF data was used to generate an elemental color map representing the amount of metal present, which is a proxy for the Ti and Zn ceramic fillers. The results demonstrate that the ceramic fillers were uniformly distributed (Figure 4).

### 3.2. Thermal Characterization

#### 3.2.1. Thermal Phase Behavior

Thermal characterization provides insight into the phase transitions and stability of a material. This is especially useful when the filaments are going to be used for FFF, which requires heating past the material’s glass transition temperature or melting point in a nozzle in order to have a suitable flow and then sufficiently cool on a heating bed so a rigid and final product can be obtained. While the binding material, in this case PLA, can be used for 3D-printing, if the resulting composite changes the phase transitions enough or lowers the thermal stability by an appreciable amount, then it is no longer a feasible material for FFF applications.

PLA is reported to have a glass transition temperature (*T*_g_) between 55 and 65 °C and a maximum melting point (*T*_m_) of 175–180 °C in the purely l-isomer form, with a 5 °C decrease in *T*_m_ for every 1% increase of d-lactate in the polymer [40,53]. Indeed, the manufacturer reports that the neat PLA pellets have a glass transition temperature between 55 and 60 °C as well as a heat distortion temperature of 55 °C. The PLA filaments generated in this study exhibited a *T*_g_ of 62 °C and a *T*_m_ of 150 °C, suggesting that there is approximately 5% d-lactide within the PLA polymer. Calculating the percent crystallinity can be performed by integrating the heat flow through a sample during the melting transition, which provides information about the microstructure and can be related to explain macroscopic properties such as mechanical strength. It should be noted that some forms of PLA do not readily crystallize and while some research groups report the cold crystallization temperature of PLA and PLA composites, others report that no phase transition was observed at all. That is the case with all the filaments produced in this study; none of the filaments were found to possess a crystallization temperature for the experimental conditions employed in this work, even with the cooling rate of 1 °C/min.

**Figure 5:**
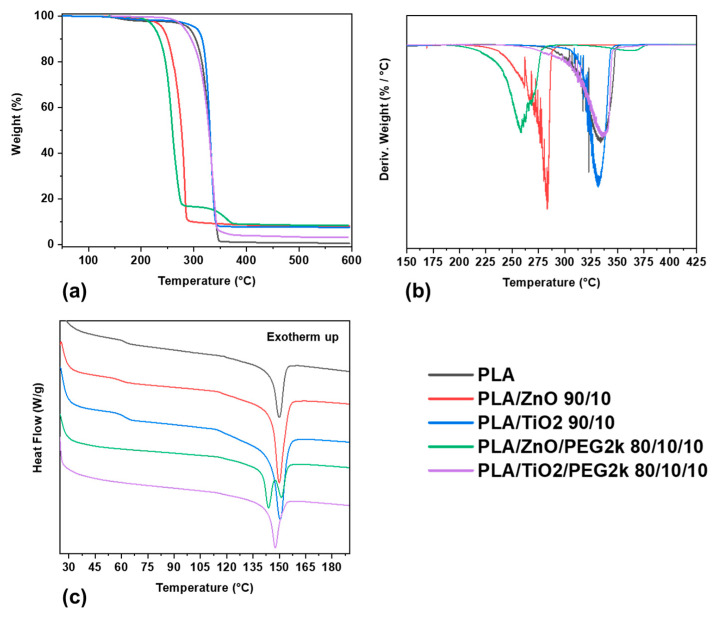
Thermograms of selected filaments: (**a**) *T*_g_A; (**b**) derivative of *T*_g_A; and (**c**) DSC (exotherm up) of the first heating curve.

Some researchers have added various fillers to PLA and altered the temperatures at which thermal phase transitions occur [22,33,39,40]. The composite filaments fabricated in this study did exhibit a slight increase in the *T*_m_ (between 5 and 7 °C), however significant changes in the *T*_m_ value were not observed as a function of the amount of the Ti or Zn ceramic filler incorporated into the material. The thermal phase transitions and percent crystallinity of the filaments fabricated in this study are listed in Table S3. Additionally, the thermograms of a selected representative sample of filaments are shown in Figure 5. Incorporation of the ceramic fillers did not alter the *T*_g_ of the filaments. Previous studies examining the thermal properties of PLA imbued with ceramic nanocomposites report conflicting results. For example, multiple research groups have shown that ZnO and TiO_2_ nanoparticles have no effect on the thermal phase behavior of PLA in the composite form [24,54,55,56]; however Buzarovska found that the *T*_g_ values increased slightly upon the incorporation of TiO_2_ nanoparticles [33], while Mallick et al. found that they could no longer detect the *T*_g_ or *T*_c_ [22], and Carrion et al. observed that increasing amounts of ZnO nanocomposites in polycarbonate/ZnO decreased the *T*_g_ [57]. The fact that the *T*_m_ and *T*_g_ of these ceramic composite materials vary across many laboratory conditions provides ever increasing evidence that additional research is required to assess the underlying micro and macroscopic effects and perform systematic batch testing on the composites as they are fabricated.

Unlike the effect of ceramics on the thermal phase behavior of PLA filaments, the incorporation of PEG significantly lowered the *T*_g_. This phenomenon has been observed before [15,36], which is bolstered by the fact that the *T*_g_ of PEG, depending on the molecular weight, can vary between −20 and −60 °C. Indeed, incorporating higher molecular weight PEG to PLA had a dramatic effect, where the composite filaments with PEG2k and PEG10k had no observable *T*_g_ in the temperature range tested (25–200 °C). Furthermore, all the ceramic composite filaments containing PEG, except for PLA/ZnO/PEG2k 80/10/10 (which had a *T*_g_ of 59 °C), did not exhibit an observable *T*_g_ in the temperature range tested. This indicates that chain mobility in the composites is higher than that in the neat filaments and that PEG behaves as a plasticizer in the system. Thus, the processability, or material fabrication, is enhanced by PEG and can support greater amounts of ceramic or other filler, as well as ensuring that fillers and plasticizers are well-dispersed throughout the PLA matrix [39].

**Figure 6:**
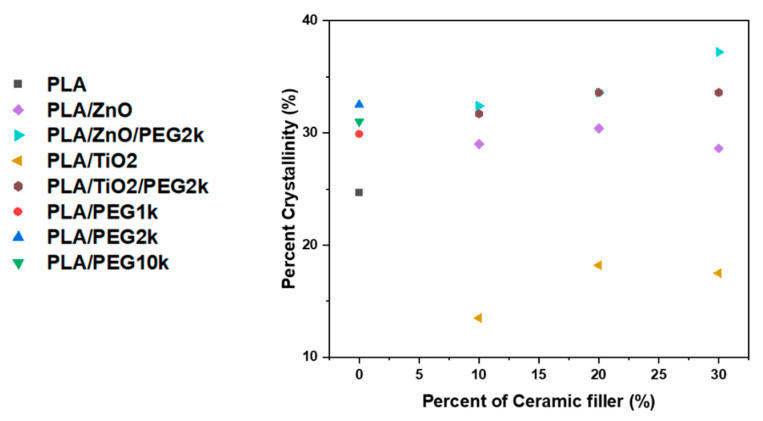
Crystallinity of filaments.

Although our results revealed little difference between the *T*_m_ of the composites and pure PLA filaments, integration of the DSC thermograms revealed that significant changes in PLA crystallinity had occurred (Figure 6; Table SI3). Compared to the PLA filament, which was 25% crystalline, incorporating the polymer plasticizer PEG into PLA led to an increase in crystallinity. Inclusion of 10 wt% PEG1k, PEG2k, or PEG10k yielded filaments with 30%, 32%, and 31% crystalline PLA, respectively. Additionally, the ternary composite filaments comprising PLA, ceramics, and PEG exhibited double melting peaks (see Figure 5c). This behavior was observed for all the ZnO ternary composite filaments and only at higher TiO_2_ concentrations (20 wt% and 30wt%), where the two peaks were at most 10 °C apart.

The addition of ZnO increased the crystalline portion of PLA. Increasing the amount of ZnO in the composite from 10 to 30 wt% did not have a major effect on crystallinity, where addition of 10, 20, and 30 wt% ZnO produced filaments that were each ~30% crystalline. This indicates that ZnO acts as a nucleating agent for the PLA chains. A similar phenomenon has been reported in literature evaluating the crystallinity of PLA/ZnO composites [54,56,58]. TiO_2_ had the opposite effect on crystallinity. Addition of 10 wt% TiO_2_ yielded a 14% crystalline PLA filament. Increasing the TiO_2_ content to 20 and 30 wt% resulted in filaments that were ~18% crystalline. Again, these results are inconsistent with those reported for nanocomposites, where it was found that the crystallinity of PLA/TiO_2_ composites dramatically increases compared to neat PLA [33]. Inclusion of 10% PEG2k increased the PLA crystallinity from 3 to 5% in each of the ZnO-PLA composite (10–30 wt% ZnO). A similar trend is observed for the inclusion of PEG2k in the PLA/TiO_2_ composites, however the increase in crystallinity was much more dramatic. That stated, while the composite filaments that incorporated PEG, ZnO, or TiO_2_ displayed either an increase or decrease in the degree of crystallinity as compared to that of the PLA filaments, morphological analysis was not performed and thus it is not certain that PEG, ZnO, or TiO_2_ is actually promoting crystal growth or changing the crystalline lamellar size.

#### 3.2.2. Thermal Stability

**Figure 7:**
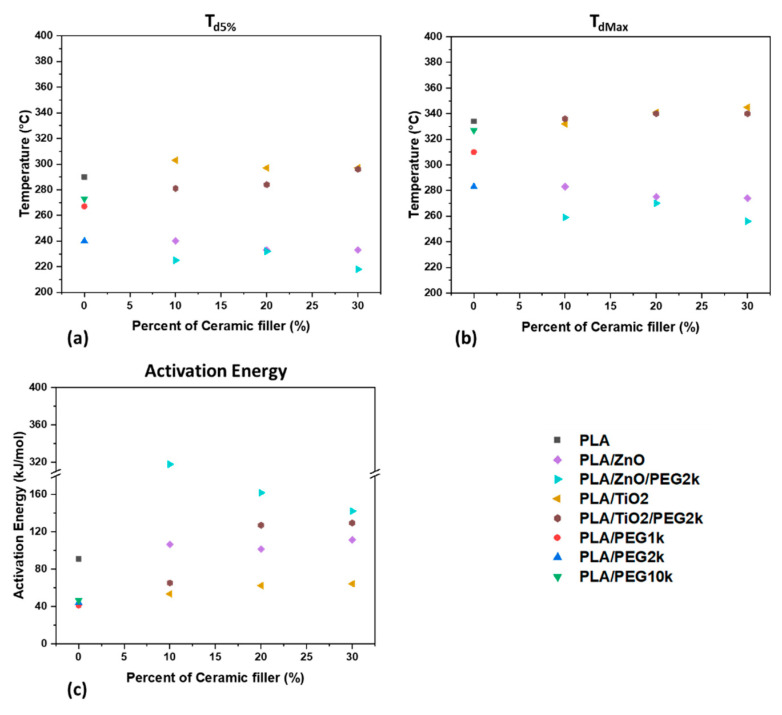
The thermal properties of the composite filaments: (**a**) the onset of thermal degradation (*T*_d5%_); (**b**) the decomposition temperature (*T*_dMax_); and (**c**) the activation energy.

Measures of thermal stability, *T*_d5%_ and *T*_dMax_, characterize the temperatures at which a material will degrade and decompose. It should be noted that these temperatures can be dependent on the heating rate of the *T*_g_A experiment; higher heating rates will result in higher values for the thermal stability. This is mainly thought to be due to the delay in the temperature a material experiences compared to the temperature of the surrounding environment. Five different heating rates were used during testing (5, 8, 10, 13, and 15 °C/min) and a shift towards higher degradation and decomposition temperatures was observed for faster heating rates. A similar trend was found for all the filaments that the *T*_d5%_ of 15 °C/min was approximately 20 °C higher than the *T*_d5%_ at 5 °C/min. To err on the side of caution and provide data that can be used for future work by others, the values for *T*_d5%_ and *T*_dMax_ presented in Figure 7a,b and Table SI4 are taken from experiments using a heating rate of 5 °C/min.

With regard to the pyrolysis kinetic parameters, previous researchers have noted that the activation energy and pre-exponential factor can also be dependent on the heating rate in a similar manner to the thermal stability. Additionally, it has been observed that at higher heating rates (e.g., *β* > 30 °C/min for polyethylene terephthalate and *β* > 80 °C/min for polystyrene [46,59]), the activation energy becomes nearly constant. Because of this, the pyrolysis kinetic parameters were determined from the highest heating rate tested (*β* = 15 °C/min). When using Equation (1), it is important to test for which reaction model produces the best fit to the data. Eight different models were evaluated on the PLA filament, spanning mechanisms that cover chemical reactions, diffusion-controlled reactions, and phase boundary reactions [45,46,47]. The models were plotted from near the beginning of the *T*_g_A experiment until the T_dMax_, upon which almost every single model showed a sharp break, which can be assumed to follow a different pyrolysis mechanism. For the PLA filament, the best fitting models were the first-order chemical reaction

(g(α) = −ln (1 − α)) and one-dimensional diffusion parabolic law (g (α) = α2), with the diffusion model fitting slightly better. Because of this, it was assumed that the pyrolysis mechanism follows a one-dimensional diffusion parabolic law reaction until decomposition and all the other filaments were evaluated in this manner. Thus, unlike the thermal stability values, which were taken using a heating rate of *β* = 5 °C/min, the pyrolysis kinetic parameters were determined using a heating rate of *β* = 15 °C/min. The calculated thermal activation energies for the filaments are presented in Figure 7c and Table SI4.

For the thermal stability of the filaments, the trends as observed in Figure 7 are that the addition of PEG results in a composite material that degrades and decomposes earlier. This can be observed comparing PLA to PLA/PEG as well as comparing PLA/ceramics to PLA/ceramics/PEG2k in Figure 7. This behavior has been exhibited in PLA/PEG mixtures in previous studies [36,60], with explanations spanning from neat PEG, which exhibits a lower thermal stability affecting the overall composite, to PEG lowering the crystallinity and thus making the polymer blend easier to degrade. However, as seen in Figure 6, the PLA/PEG filaments possess a higher crystallinity than PLA filaments, therefore the former explanation may provide a more plausible insight into the physical phenomenon. Interestingly, the polymer blends with PEG1k and PEG10k demonstrated better thermal stability than PEG2k, which could indicate how plasticizer size affects the PLA matrix. It appears that increasing from small to medium-sized PEG molecules contribute towards breaking up the composite at lower thermal energies, however the trend is reversed when larger PEG molecules are incorporated. This can be inferred to mean that greater miscibility occurs with PEG at greater molecular weights.

The thermal stability of composite filaments with ceramic fillers, shown in Figure 5 and Figure 7a,b indicate that TiO_2_ provides better thermal stability than ZnO of similar composition by about 60 °C. Indeed, while TiO_2_ composite filaments had thermal stabilities near that or slightly better than PLA, the incorporation of ZnO significantly reduced the onset thermal degradation and decompositions. This phenomenon has been observed before, where incorporating TiO_2_ into PLA has led to slightly better thermal stability [24]. The opposite effect has been demonstrated by incorporating ZnO, which has decreased the *T*_d5%_ and *T*_dMax_ of PLA composites [26,51,54]. This effect can be explained by the catalytic role zinc compounds play in the transesterification reaction of lactide oligomers [61], which is further supported with Figure 7c, discussed below. It should be noted that Wang et al. and Mallick et al. found that PLA/TiO_2_ nanocomposites exhibited lower thermal stability than neat PLA, however the varied results could be due to the use of amorphous PLA and the presence of solvent, respectively [22,34].

It was found that the calculated activation energies and pre-exponential factors followed trends similar to each other. The data supports the observed thermal stabilities, where filaments with lower activation energy, which is a measure of the energy barrier for the pyrolysis reaction, degraded at lower temperatures than those with higher activation energies. Differences to this observation can be found in the ZnO composites, which can be explained with the pre-exponential factors found in Table S4. Although the activation energy for these filaments is larger than the others, the pre-exponential factor, which is a measure of collisions and reactions per unit time, are also significantly higher, with PLA/ZnO 90/10 and PLA/ZnO/PEG2k 80/10/10 having around 2 and 23 orders of magnitude above PLA, respectively.

### 3.3. Mechaniacal Characterization

Through tensile testing the composite filaments, three values were measured: the engineering stress, engineering strain, and the Young’s modulus. For relevant understanding of mechanical properties, the averaged maximum values for stress and strain were tabulated. Maximum stress values occurred after the initial linear viscoelastic region, which is useful in understanding capacity and loads that could be withstood. After the linear region, a necking phenomenon occurred and stress values undulated as strain kept monotonically increasing. The maximum strain occurred at the moment of filament breakage, which is useful in understanding the physical mechanical limits of the material. The Young’s modulus is the slope of the linear viscoelastic region and is a measure of whether a material exhibits flexibility or brittleness. All three measured mechanical properties for the composite filaments are shown in Figure 7 and Table SI5. For the processed PLA filament, the averaged maximum engineering stress and strain and Young’s modulus were 49 MPa ± 7 MPa, and 6% ± 2%, and 2314 MPa ± 300 MPa, respectively.

When ceramics were incorporated into the PLA network, the mechanical properties of the filaments became measurably different. A small decrease in maximum stress is observed upon addition of either ZnO or TiO_2_, which is mostly a constant step drop for each of the filler additions; however, a large decrease in maximum stress occurred in the PLA/TiO_2_ 70/30 sample. The addition of 10 wt% ceramic filler did not alter the maximum strain of the PLA filament, but increasing the ceramic content to 20 or 30 wt% decreased the maximum strain noticeably. At similar wt%, ZnO and TiO_2_ composites exhibited comparable maximum strain values. It has been reported that adding ceramics to polymers makes the overall material more brittle, and thus increases the Young’s modulus [35]. Yet embedding ZnO in PLA increased the Young’s modulus only slightly, with the greatest change of 15% occurring in the PLA/ZnO 70/30 composite. Interestingly, all samples containing TiO_2_ exhibited a decrease in the Young’s modulus, with no relationship between the wt% added and the magnitude of the decrease. This can be explained from the crystallinity view point; previous research showing incorporating TiO_2_ increased the brittleness also showed an increased in crystallinity [33]. Because the composite filaments with TiO_2_ exhibited a lower crystallinity, the strength decreases accordingly.

It is worth pointing out that the maximum stress is reduced when incorporating 10 wt% PEG, with the effect inversely proportional to the molecular weight of the PEG added. While the maximum stress decreased upon addition of PEG, the maximum strain was significantly increased by its incorporation. It should be noted that while PLA/PEG1k exhibited a 741% increase in strain when compared with PLA, PLA/PEG10k exhibited a 1129% increase, and PLA/PEG2k exhibited a 1661% increase. PEG is known for rendering PLA and other polymers more pliable [36], which is similarly demonstrated here for filaments. Incorporating PEG into the PLA matrix caused a reduction in the Young’s modulus, with higher molecular weight PEGs yielding lesser effects. It should be noted that in this vein, PLA/PEG1k and PLA/PEG2k composites exhibited approximately three-fold lower Young’s-modulus values than PLA filaments, whereas the value for the PLA/PEG10k composite was only slightly lower (13.5%) than that of PLA comparatively.

**Figure 8:**
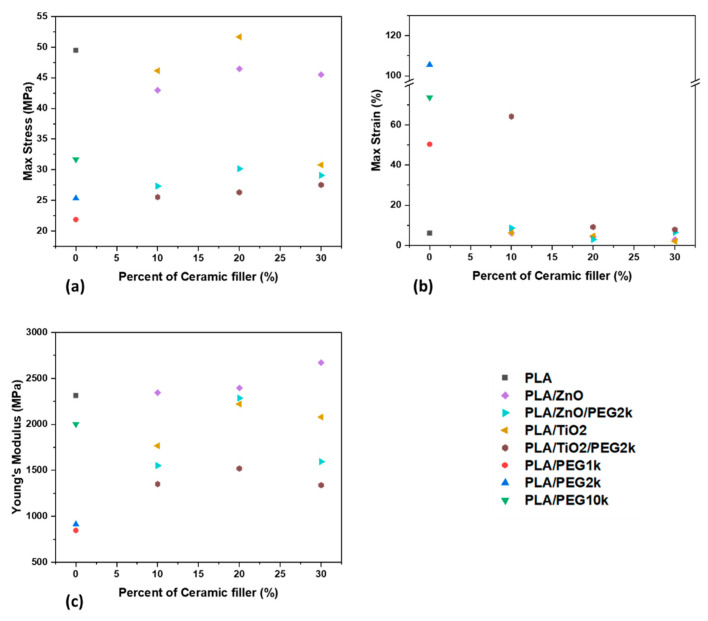
The mechanical properties of the composite filaments: (**a**) max stress; (**b**) max strain; and (**c**) Young’s modulus.

The mechanical properties of the composites with ceramic fillers and PEG are shown in Figure 8. It was observed that 10 wt% PEG2k reduced the maximum stress of all samples with ZnO and TiO_2_. Additionally, while altering the amount of ceramic filler altered the maximum stress, inclusion of 10 wt% PEG2k exhibited similar values for each sample tested, regardless of filler content. The reverse was observed with regards to maximum strain; adding PEG2k increased the elongation of the samples, with the sole exception of PLA/ZnO/PEG2k 70/20/10, which was lower than PLA/ZnO 80/20. The largest change was observed in PLA/TiO_2_/PEG2k 80/10/10, which demonstrated a 938% increase compared to PLA/TiO_2_ 90/10. Similar to maximum stress, adding PEG2k to the composite resulted in a reduction in the Young’s modulus for all the samples and like the maximum stress of the samples, altering the amount of ceramic filler does not significantly change the Young’s modulus in the filaments with PEG2k incorporated, with the only exception being PLA/ZnO/PEG2k 70/20/10. This sample showed minor differences compared to PLA/ZnO 80/20, suggesting that the stiffness does not change drastically.

### 3.4. Antimicrobial Efficacy

**Figure 9:**
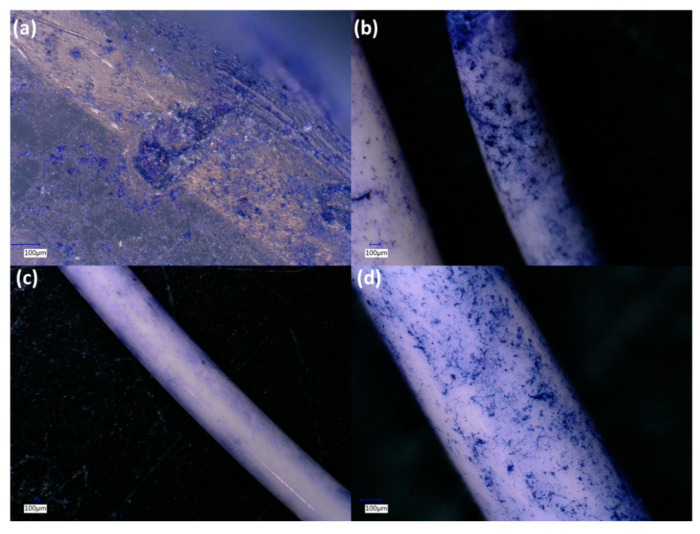
Microscopy images of stained composite filaments that were incubated in soil for a month: (**a**) PLA; (**b**) PLA/TiO_2_ 90/10 with gelatin; (**c**) PLA/ZnO 90/10; and (**d**) PLA/ZnO 90/10 with gelatin.

Microbial activity (alteration of the filament surface) was most prominent on pure PLA filaments, where abundant colonial and filamentous growth patterns were observed. Interestingly, the surface of the PLA filament incubated in soil exhibited areas where relatively large “flakes” (50–100 microns in diameter) had sloughed from the surface (e.g., Figure 9a). These regions of “flaking” were typically associated with robust microbial colonization. Inclusion of TiO_2_ and, especially, ZnO ceramics into the PLA filaments reduced the amount of visible microbial colonization and surface alteration (Figure 9a vs. Figure 9b,c). The addition of PEG did not affect these properties; thus, it can be used purely to tune the physical material properties of the filaments without affecting the biodegradability of the polymers. The addition of 10 wt% gelatin to the soils did not have a noticeable effect on microbial colonization patterns or pitting/flaking on the filament surfaces (data not shown).

The PLA, PLA/TiO_2_ 90/10, and PLA/ZnO 90/10 filaments were analyzed to find the regions with the greatest amount of pitting so that the degradation could be measured on a per area basis. Pitting is a removal of the surface layer, so a quantitative determination could be performed by finding the ratio of area removed against the surface area measured. In the most damaged area, pits covered 1.30% of the PLA filament surface, compared to 0.40% and 0.34% of the PLA/TiO_2_ 90/10 and PLA/ZnO 90/10 filament surfaces, respectively. Additionally, the largest pits found measured approximately 20,000 µm^2^ for PLA, compared to 7000 µm^2^ and 2000 µm^2^ for the PLA/TiO_2_ 90/10 and PLA/ZnO 90/10, respectively. Thus, adding the ceramics not only reduced the amount of visible microbial colonization and surface alteration, but also had a measured order of magnitude less microbial degradation as well.

## 4. Conclusions

Antimicrobial composites have been heavily researched and successfully transitioned into commercial products, yet the question of whether their mechanical properties can be tuned while engineering additional multifunctionality had not been addressed for 3D-printing applications before this study. We demonstrate that by using a solvent treatment method for filament feedstock fabrication, homogenous composites can be developed for 3D-printing applications. High weight percent loading (10–30 wt%) of TiO_2_ and ZnO ceramic fillers into PLA filaments was achieved to imbue antimicrobial characteristics, and PEG was added to control the mechanical properties as well as allow pathways for further biological modifications.

By fabricating the filaments using the process described in this study, homogeneous materials were created that can be used for FFF. Thermal phase behavior and stability demonstrated that the composite filaments will soften during the 3D-printing process and survive at elevated temperature regimes. Indeed, all the composite filaments are stable up to 200 °C, which is suitable for FFF regimes. Furthermore, the filaments with TiO_2_ resulted in being more thermally stable than those with ZnO, where a 60 °C difference was observed. Additionally, the mechanical behaviors can be tuned based on ceramic filler loading and types and via the inclusion of PEG. This is demonstrated by the inclusion of 10 wt% PEG generally reducing the maximum stress of a by 35–50%, while dramatically increasing and decreasing the maximum elongation and Young’s modulus, respectively, in some cases. Finally, incorporating ceramic fillers significantly reduced pitting and degradation on PLA filament surfaces due to microbial activity. PEG did not affect the antimicrobial properties of the ceramic/PLA composites, so it can be added solely to alter the physical material properties. Our study thus expands the range of functional materials for FFF and advanced manufacturing.

## Figures and Tables

**Table 1:** List of the composite filaments made in this study and their designations.

Sample	Material
PLA	100 wt% PLA
PLA/PEG1k 90/10	90 wt% PLA, 10 wt% PEG (MW, 1 kDa)
PLA/PEG2k 90/10	90 wt% PLA, 10 wt% PEG (MW, 2 kDa)
PLA/PEG10k 90/10	90 wt% PLA, 10 wt% PEG (MW, 10 kDa)
PLA/ZnO 90/10	90 wt% PLA, 10 wt% ZnO
PLA/ZnO 80/20	80 wt% PLA, 20 wt% ZnO
PLA/ZnO 70/30	70 wt% PLA, 30 wt% ZnO
PLA/ZnO/PEG2k 80/10/10	80 wt% PLA, 10 wt% ZnO, 10 wt% PEG (MW, 2 kDa)
PLA/ZnO/PEG2k 70/20/10	70 wt% PLA, 20 wt% ZnO, 10 wt% PEG (MW, 2 kDa)
PLA/ZnO/PEG2k 60/30/10	60 wt% PLA, 30 wt% ZnO, 10 wt% PEG (MW, 2 kDa)
PLA/TiO_2_ 90/10	90 wt% PLA, 10 wt% TiO_2_
PLA/TiO_2_ 80/20	80 wt% PLA, 20 wt% TiO_2_
PLA/TiO_2_ 70/30	70 wt% PLA, 30 wt% TiO_2_
PLA/TiO_2_/PEG2k 80/10/10	80 wt% PLA, 10 wt% TiO_2_, 10 wt% PEG (MW, 2 kDa)
PLA/TiO_2_/PEG2k 70/20/10	70 wt% PLA, 20 wt% TiO_2_, 10 wt% PEG (MW, 2 kDa)
PLA/TiO_2_/PEG2k 60/30/10	60 wt% PLA, 30 wt% TiO_2_, 10 wt% PEG (MW, 2 kDa)

**Table 2:** FTIR peaks associated with the spectra of PLA in Figure 3.

Peak Number	Wavenumber (cm^−1^)	Vibrational Mode
1	1080, 1187	C-O stretching
2	1361	Symmetric -CH_3_ bending
3	1452	Asymmetric -CH_3_ bending
4	1746	C = O stretching
5	2946	Asymmetric -CH_3_ stretching
6	2995	Symmetric -CH_3_ stretching

## Data Availability

Data are contained within the article and supplementary information. Additional data may be provided upon request.

## Supplementary Materials

The following are available online at https://www.mdpi.com/2073-4360/13/4/580/s1, Table S1: Average and standard deviations of D10, D50, and D90 for each procedure of ZnO powder; Table S2: Average and standard deviations of D10, D50, and D90 for each procedure of TiO_2_ powder; Table S3: Thermal phase transitions and crystallinity of the composite filaments; Table S4: Thermal stability of the composite filaments (decomposition values take from 5 °C/min and thermal kinetic parameters taken from 15 °C/min); and Table S5: Mechanical properties of the composite filaments.
