# Supplementary material for: Development of Antimicrobial PLA Composites for Fused Filament Fabrication
Source: Polymers (Basel). 2021 Feb 15;13(4):580. doi: 10.3390/polym13040580 (PMC7918987; doi:10.3390/polym13040580)
Supplement: Supplementary file 1 [file polymers-13-00580-s001.pdf]

Supplemental Information

Table SI1: Thermal phase transitions and crystallinity of the composite filaments

Sample	T_g (℃)	T_m (℃)	ΔH_m (J/g)	X_c (%)
PLA	60	145	23.04	25
PLA/ZnO 90/10	59	152	24.32	29
PLA/ZnO 80/20	59	152	22.62	30
PLA/ZnO 70/30	59	152	18.63	29
PLA/TiO2 90/10	60	149	11.35	14
PLA/TiO2 80/20	61	150	13.58	18
PLA/TiO2 70/30	61	149	11.39	17
PLA/PEG1k 90/10	52	153	25.02	30
PLA/PEG2k 90/10	-	151	27.22	32
PLA/PEG10k 90/10	-	151	25.98	31
PLA/ZnO/PEG2k 80/10/10	59	152	24.13	32
PLA/ZnO/PEG2k 70/20/10	-	152	21.92	34
PLA/ZnO/PEG2k 60/30/10	-	152	20.76	37
PLA/TiO2/PEG2k 80/10/10	-	151	23.59	32
PLA/TiO2/PEG2k 70/20/10	-	152	21.88	34
PLA/TiO2/PEG2k 60/30/10	-	151	18.78	34

Table SI2: Thermal stability of the composite filaments (decomposition values take from 5℃/min and thermal kinetic parameters taken from 15℃/min)

Sample	$T_{d5\%}$ (℃)	T_{dMax} (℃)	E_a (kJ/mol)	A (min^{-1})
PLA	290	334	91.0604	$3.51*10^{5}$
PLA/ZnO 90/10	240	283	106.4672	$4.81*10^{7}$
PLA/ZnO 80/20	233	275	101.5866	$2.34*10^{8}$
PLA/ZnO 70/30	233	274	111.3312	$1.82*10^{9}$
PLA/TiO2 90/10	303	332	53.44145	$7.26*10^{1}$
PLA/TiO2 80/20	297	341	62.36291	$1.32*10^{3}$
PLA/TiO2 70/30	297	345	64.21288	$2.27*10^{3}$
PLA/PEG1k 90/10	267	310	41.02541	$4.05*10^{-1}$
PLA/PEG2k 90/10	240	283	44.06685	$2.42*10^{0}$
PLA/PEG10k 90/10	273	327	46.33837	$1.20*10^{1}$
PLA/ZnO/PEG2k 80/10/10	225	259	317.722	$6.04*10^{28}$
PLA/ZnO/PEG2k 70/20/10	232	270	161.9581	$7.74*10^{13}$
PLA/ZnO/PEG2k 60/30/10	218	256	142.2528	$9.74*10^{11}$
PLA/TiO2/PEG2k 80/10/10	281	336	65.18235	$2.52*10^{3}$
PLA/TiO2/PEG2k 70/20/10	284	340	127.1121	$1.40*10^{9}$
PLA/TiO2/PEG2k 60/30/10	296	340	129.4734	$1.78*10^{9}$

Table SI3: Mechanical properties of the composite filaments

Sample	Max Stress (MPa)	Max Strain (%)	Young's Modulus (MPa)
PLA	49.5	6.00	2314
PLA/ZnO 90/10	43.0	6.18	2345
PLA/ZnO 80/20	46.5	4.30	2397
PLA/ZnO 70/30	45.5	2.78	2671
PLA/TiO2 90/10	46.2	6.19	1767
PLA/TiO2 80/20	51.7	4.67	2221
PLA/TiO2 70/30	30.8	2.01	2080
PLA/PEG1k 90/10	21.9	50.40	846
PLA/PEG2k 90/10	25.3	105.53	912
PLA/PEG10k 90/10	31.7	73.65	2002
PLA/ZnO/PEG2k 80/10/10	27.3	8.62	1551
PLA/ZnO/PEG2k 70/20/10	30.2	2.95	2286
PLA/ZnO/PEG2k 60/30/10	29.1	6.40	1595
PLA/TiO2/PEG2k 80/10/10	25.5	64.24	1351
PLA/TiO2/PEG2k 70/20/10	26.3	9.15	1520
PLA/TiO2/PEG2k 60/30/10	27.5	7.88	1338
